# Centrosome instability: when good centrosomes go bad

**DOI:** 10.1007/s00018-021-03928-1

**Published:** 2021-09-02

**Authors:** John M. Ryniawec, Gregory C. Rogers

**Affiliations:** University of Arizona Cancer Center, University of Arizona, 1515 N. Campbell Ave., Tucson, AZ, 85724, USA

## Abstract

The centrosome is a tiny cytoplasmic organelle that organizes and constructs massive molecular machines to coordinate diverse cellular processes. Due to its many roles during both interphase and mitosis, maintaining centrosome homeostasis is essential to normal health and development. Centrosome instability, divergence from normal centrosome number and structure, is a common pathognomonic cellular state tightly associated with cancers and other genetic diseases. As novel connections are investigated linking the centrosome to disease, it is critical to understand the breadth of centrosome functions to inspire discovery. In this review, we provide an introduction to normal centrosome function and highlight recent discoveries that link centrosome instability to specific disease states.

## A historical perspective

Since the advent of cell theory by Schleiden and Schwann nearly 200 years ago, the idea of self-replicating biological units and, thus, the basic requirement for cell division, have captivated scientists. In 1887, advances in cytology allowed for the discovery of karyokinetic division – equal segregation of genomic material into two daughter cells – in mitotic nematode embryos by Theodor Boveri and in meiotic worm eggs by Edouardo Van Beneden (called pseudokaryokinesis due to its reductional nature) [1–3]. Independently, but concurrently, they discovered that condensed chromosomes are aligned between a bipolar filamentous array and, subsequently, divided into two daughter cells. In his original study, Boveri discussed the organization of this astral array, defining the centrosome as the center of each pole. His findings led him to believe that the centrosome is “the true division organ of the cell, it mediates the nuclear and cellular division” [1]. Over the next 30 years, Boveri made several seminal discoveries regarding the centrosome and became the father of an enduring field of study that would expand well beyond cell division [3].

Although the centrosome is involved in numerous biological processes, most historical research focused on the role of the centrosome during cell division. In 1890, David von Hansemann observed cancer cells undergoing multipolar mitosis, as opposed to a typical bipolar mitosis [4, 5] This discovery led Boveri to hypothesize that cells containing supernumerary centrosomes (more than two) would generate a multipolar spindle during mitosis [3, 5]. While performing his classic dispermy experiments, Boveri noted that sea urchin eggs fertilized by two sperm also produced multipolar spindles. In many animals, including sea urchins, eggs normally lack centrosomes but acquire one from the sperm upon fertilization. When two sperm enter an egg, the resultant zygote contains not only double the number of chromosomes but double the number of centrosomes as well. Embryos with an abnormally elevated number of centrosomes can then undergo a multipolar mitosis accompanied by unequal chromosome segregation [3, 5–7]. Together with his observations that dispermic zygotes had “malfunctions”, Boveri postulated in 1914 that a supernumerary centrosome-induced multipolar mitosis can directly generate aneuploid daughter cells and, potentially, cancer [3, 5, 8]. Since then, generations of scientists have cultivated centrosome biology into a field of its own and now explore a myriad of questions from how cells control centrosome biogenesis to understanding centrosome function during normal development and its dysregulation in disease.

Centrosome number is governed by the centriole, the duplicating element at the core of the organelle [9]. The centriole was first described in 1900 by none other than Boveri [3, 10]. In 1895, he reported a prominent, densely-stained granule at the center of the centrosome, but it was not until five years later that he described its duplicating nature and named it the centriole [3, 10, 11]. While observing dividing sea urchin embryos, Boveri reported the separation of centrosomes during the 2-cell stage, prior to the second cleavage event. Since he knew that sperm contributed only one centrosome during fertilization, he concluded that the centrosome needed to duplicate prior to each round of mitosis [10]. Modern studies have corroborated his interpretation, centrioles in fact duplicate once per cell cycle ensuring that cells have only two centrosomes as they enter mitosis. Each centrosome then organizes a single pole of the mitotic spindle to ensure that a bipolar structure is produced [9]. The need for duplication was obvious to Boveri, but not to others of his time [3]. Without duplication of centrosomes (or the genome), developing organisms would run out of materials essential for the execution of cellular divisions before they fully matured. His conviction to this idea carved his place as the father of the centrosome field, instead of his contemporaries. And although Boveri first described the cyclical nature of centrosome duplication in 1900, it was not until the modern age of molecular biology and genetic manipulation that we truly began to understand the mechanisms that underly this cycle [1, 3, 9, 10].

**Fig. 1:**
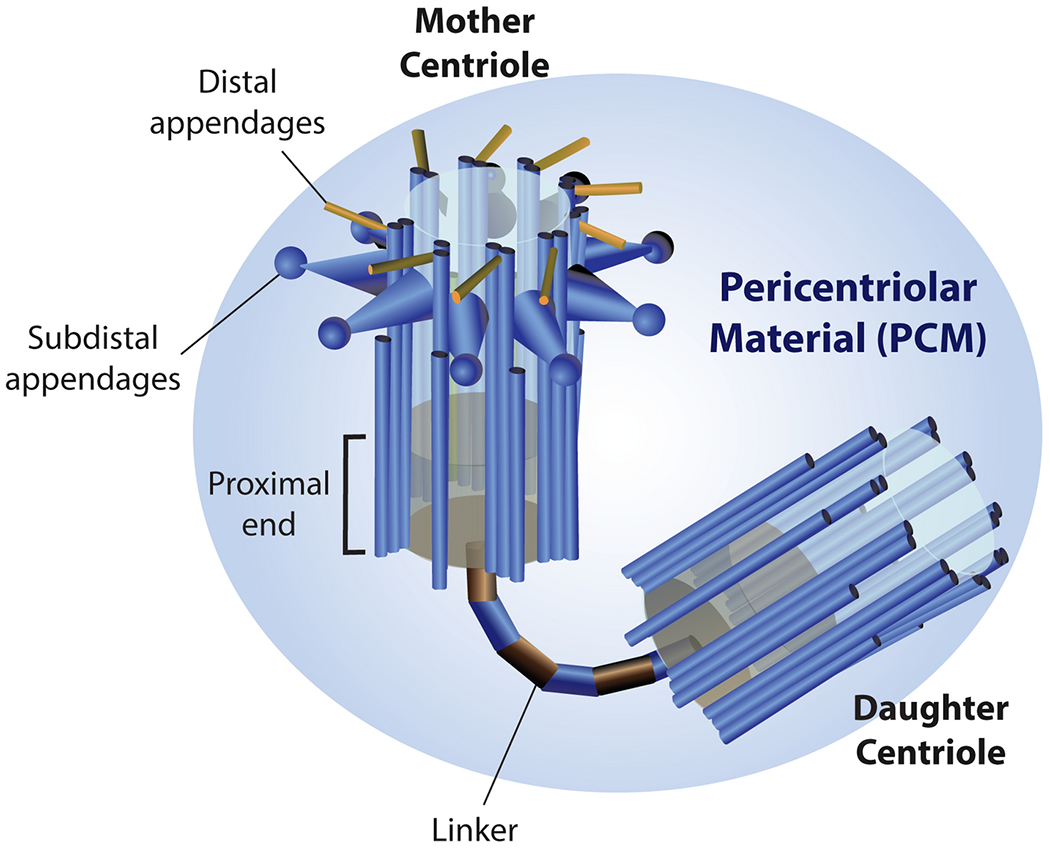
Centrosomes are microtubule-organizing and nucleating centers of cells. Cartoon of the vertebrate centrosome consisting of a mother-daughter centriole pair and surrounded by an organized shell of pericentriolar material (PCM). A mother centriole is crowned with subdistal and distal appendages, distinguishing it from its daughter centriole. A fibrous protein linker connects the proximal ends of the centriole pair, later removed prior to mitotic entry. The PCM acts as a scaffold for the γ-tubulin ring complex, allowing the organelle to nucleate microtubule growth.

The centriole itself is an important cellular organelle; not only does it control centrosome copy number, but centriole structure is critical for centrosome function and the assembly of other microtubule-based protein machines, like cilia (Fig. 1) [12]. The first transmission electron micrograph of the centriole was published in 1954, when Fawcett and Porter observed the basal bodies of cilia in multiciliated cells (although they did not identify these structures as centrioles, but rather as “basal corpuscles”) [13]. Therefore, the first centriole micrographs are commonly attributed to de Harven and Bernhard in 1956, when the authors characterized centrioles in the cells of newts, chicken, mice, rats, and human cancer tissues [14]. This study described the evolutionarily conserved barrel-shape of the centriole and established that the centriole is composed of microtubule bundles arranged in 9-fold radial symmetry [13–15]. As electron microscopy techniques improved, so did our appreciation of the diversity of centriole structure. While most mammalian centrioles are made of triplet microtubule bundles, animals such as *Caenorhabditis elegans* and *Drosophila melanogaster* can produce centrioles with singlet and doublet microtubules [16]. Furthermore, surveys of different *Drosophila* tissues revealed diverse centriole architecture, even within an individual. Some *Drosophila* tissues have long centrioles composed of microtubule triplets, while others have short centrioles made of a mixture of microtubule doublets and singlets [17]. It is this tissue-specific structural diversity within an individual that highlights the complex nature of centriole assembly. Indeed, this diversity reveals that centrioles are not constructed from a singular blueprint. Instead, the molecular mechanisms underlying centriole architecture must be plastic, responding to contextual cues to build the correct centriole for the cell’s needs.

## The centrosome: a tiny organelle with big responsibilities

Nearly 140 years since the term ‘centrosome’ was used, we continue to discover new functions and find new ways that centrosomes contribute to development and homeostasis. No matter how many functions we identify, one fact remains true: the central role of the centrosome is to construct specialized microtubule-based assemblies . Centrosomes are the major microtubule-organizing center (MTOC) in most animal cells and are composed of a pair of centrioles surrounded by an organized proteinaceous shell called the pericentriolar material (PCM) [18]. The PCM meshwork essentially acts as a platform for docking γ-tubulin ring complexes, which facilitate microtubule polymerization [19]. Thus, centrosomes act as a microtubule hub to facilitate mitotic spindle assembly, generate specialized microtubule networks, and establish polarity within cells. Additionally, centrosomes can convert to basal bodies, whereby a centriole docks at the plasma membrane and grows a microtubule projection, called the axoneme, that acts as the central structure of cilia and flagella. These specialized organelles generate cellular motility, extracellular flow, and sense the external environment [20, 21]. Below, we present an overview of centrosome functions.

## Functions of the interphase centrosome

**Fig. 2:**
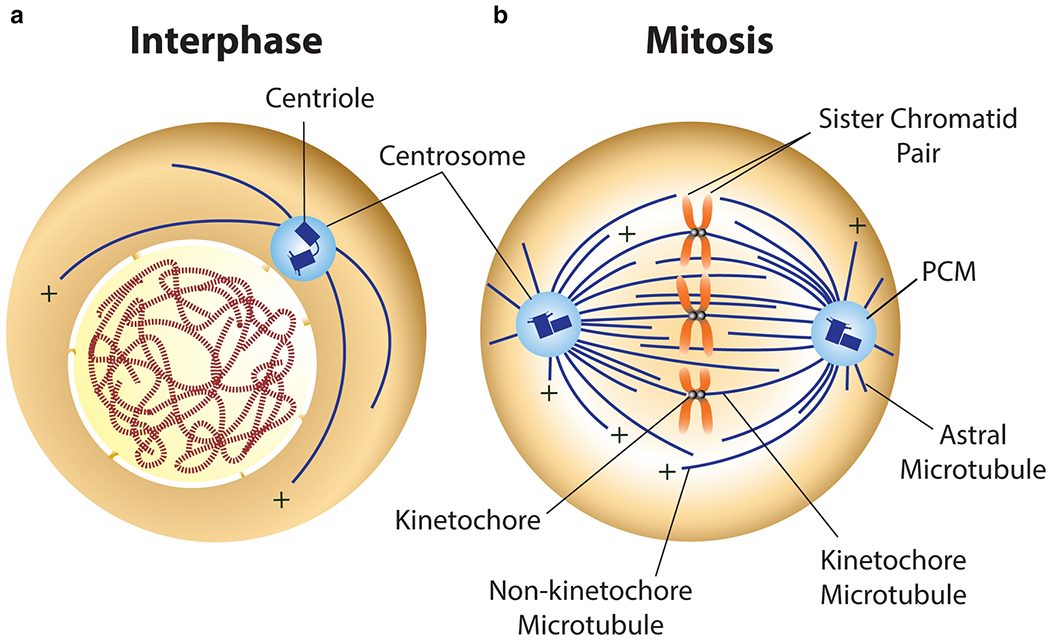
Centrosomes produce microtubule-based machines by nucleating microtubules within the pericentriolar material (PCM). Growing microtubule plus (+)-ends radiate away from the centrosome, establishing an inherent polarity. **a** During most of interphase of the cell cycle, cells contain a single centrosome tethered to the nucleus. The interphase microtubule is essential for vesicle trafficking and organelle positioning. **b** During mitosis, cells contain two centrosomes. Each centrosome controls spindle shape by organizing a distinct pole of the mitotic spindle. Spindle shape is important for the accurate segregation of chromosomes.

Although the most enigmatic function of the centrosome is construction of the mitotic spindle, the centrosome’s roles in interphase cells have become exceedingly evident (Fig. 2). In a stereotypical mammalian cell, a single centrosome is anchored near the nuclear envelope. This orients microtubules so that their plus (+)-ends grow away from the centrosome and towards the cell’s periphery. The inherent polarity is used by cells to generate and maintain cellular polarity, organize the cytoplasm by positioning organelles, and traffic membranous and protein cargo towards or away from the nucleus. Other cell types, such as neurons and epithelial cells, rely heavily on the centrosome for their cellular morphology and specialized functions [22]. In these cells, centrosomes can generate microtubules which are reorganized within neuronal processes or arranged as parallel arrays along lateral epithelial surfaces [10]. Not surprisingly, Boveri hypothesized in 1887 that the centrosome is an enduring and permanent cellular organelle, not just a transient structure during mitosis [1, 3]. The variety of centrosome functions certainly validates his theory.

Due to its polarized nature, the interphase microtubule array created by the centrosome provides a roadmap for the directional movement of microtubule-based motor proteins throughout the cell [18, 22, 23]. Kinesin motor proteins that transport organelles and vesicles are plus (+)-end directed and processive, meaning they are able to make long excursions along microtubules and towards the cell periphery (anterograde transport) [23]. Kinesins power the movement of a variety of intracellular cargo (including endosomal vesicles and lysosomes) through their interactions with cargo receptors (Gadkin:AP-1 or SKIP:Arl8, respectively) [23–25]. Conversely, cytoplasmic dynein motors move toward the minus (−)-ends of microtubules and drive the retrograde transport of subcellular cargoes [26]. Since microtubule minus-ends are anchored at the centrosome, which is typically tethered to the nucleus, cytoplasmic dynein moves cargo towards the nucleus and maintains the Golgi apparatus’ perinuclear position [27].

One of the most striking examples of this biology is the trafficking of melanosomes – specialized vesicles containing the pigment protein melanin [23, 28]. Studies in *Xenopus* and zebrafish revealed that upon stimulation with cAMP-producing hormones, perinuclear melanosomes move towards the periphery of a melanophore cell [29, 30]. Conversely, reducing cAMP levels results in retrograde movement of melanosomes towards the centrosome [30]. Subsequent *in vivo* and *in vitro* studies confirmed that changing the balance of motor activity regulates the directional transport along centrosome-derived microtubule arrays [31, 32]. These studies exemplify how cells can dynamically change their use of centrosome-derived microtubule arrays to respond to diverse stimuli. Importantly, the trafficking of melanosomes allows pigment producing cells to protect underlying skin stem cells from UV damage [28].

To establish this cellular roadmap, the centrosome is linked to the nuclear envelope which allows it to generate a polarized microtubule array that extends towards the cellular periphery. Disrupting this linkage can result in improper nuclear positioning, chromosome segregation defects, and abnormal cellular morphology [33–35]. Nuclear tethering is a microtubule-dependent process which, in turn, helps position the nucleus [36–38]. While there are multiple mechanisms in mammalian cells that link centrosomal microtubules to the nuclear periphery [39], conserved mechanisms of centrosome-nuclear tethering rely on cytoplasmic dynein anchored on the outer nuclear membrane [40].

First described in *C. elegans*, cytoplasmic dynein is tethered to the nucleus through an interaction with the LINC (LInker of Nucleoskeleton and Cytoskeleton) complex [34]. As its name implies, the LINC complex is a molecular bridge across the nuclear membrane that mechanically links the nuclear lamina, chromosomes, and chromatin-binding proteins in the nucleus with the cytoplasmic cytoskeleton (actin, microtubules, and intermediate filaments) [41–43]. In *C. elegans*, the cytoplasmic LINC protein ZYG-12, which is embedded in the outer nuclear membrane, interacts with the dynein light chain DLI-1 to tether cytoplasmic dynein to the nucleus [35, 44]. Nuclear-anchored dynein then pulls on centrosomal microtubules and positions the centrosome near the nucleus [43].

The polarity provided by the interphase microtubule array also assists in building the trans-Golgi network [27, 46–48]. During mitosis, the Golgi apparatus disassembles into vesicles and must reform at the start of the next cell cycle. Although the Golgi apparatus can independently form cisternae, centrosomal microtubules are required to stack the cisternae into the ribbons that are stereotypical of this large organelle. Fascinatingly, like the centrosome, the Golgi apparatus also acts as a MTOC capable of nucleating its own population of microtubules, mediated by CLASPs and AKAP350 [49, 50]. Cross-linking of Golgi-derived microtubules with centrosomal microtubules assists in the membrane fusion necessary to organize the Golgi ribbons into stacks [46, 51]. Moreover, these interactions establish a directional nucleus-centrosome-Golgi axis that creates a higher order polarity within the cell. Notably, the axis points toward the leading edge of migrating cells and, when disrupted, interferes with directional cell migration [27, 48, 51–54]. Furthermore, the nucleus-centrosome-Golgi axis points towards lumens in tissues and cultured organoids, allowing cells to make use of this established apico-basal polarity for trafficking events [47, 53]. Meaning, without the ability to establish a nucleus-centrosome-Golgi axis, cells have reduced secretion and exocytosis [51]. Surprisingly, recent evidence suggests that Golgi organization by centrosomal MTOCs appears to be restricted to G1-phase and early mitosis in cultured cells [54]. The cell cycle restriction of the nucleus-centrosome-Golgi axis may suggest a stronger role in trafficking within non-proliferating, differentiated cell-types than in tissue culture cells.

Specialized cell-types further co-opt centrosome-associated polarity cues to direct polarized secretion [47]. For example, upon antigen stimulation, lymphocytes quickly repolarize their centrosome-Golgi axis toward the developing immunological synapse – the site where a lymphocyte and antigen-presenting cell makes contact [55]. This is accomplished by dynein anchored at the immunological synapse which grabs centrosomal microtubules and generates a pulling force towards this site, thereby repositioning the centrosome [56, 57]. Upon arriving at the immunological synapse, the mother centriole transiently docks with the plasma membrane helping to reposition the microtubule cytoskeleton [58, 59]. The newly generated cytoskeletal hub helps to strengthen the synapse and ensures the directed effector functions of the lymphocyte. T-cells unable to polarize their centrosome to the immunological synapse cannot sustain downstream signaling and, as a result, are unable to continue cytokine production, cytolytic killing, or B cell co-stimulation [56, 60, 61].

Developing neurons, another specialized cell-type, use centrosome-derived microtubules to direct axon extension [47, 62]. Dissociated neurons cultured *in vitro* project multiple neurites before selecting one to elongate and become an axon [63]. Early studies showed that the centrosome and Golgi polarize towards the selected axon prior to its extension [64]. Additionally, centrosome-derived microtubules are essential for the elongation process [65, 66]. Although it cannot be ruled out that the centrosome is merely responding to polarity cues, some have suggested that the positioning of the centrosome selects the axon. Further, centrosome polarization is associated with neuronal migration in the developing cortex [62]. However, the necessity for the centrosome in this process has not been experimentally disassociated from its role in neuronal delamination during mitosis, which may be a prerequisite step for neuronal migration.

Finally, the centriole plays another role in interphase cells by acting as the basal body for cilia and flagella. Cilia and flagella are organelles that protrude from the main cell body and are composed of a long microtubule-based axoneme sheathed in plasma membrane [20, 21]. Flagella are commonly used to propel cells, such as sperm. Cilia, on the other hand, come in two varieties: motile and non-motile. In *Tetrahymena*, motile cilia line the outside of the cell and coordinate their beating to promote cell locomotion. In mammals, motile cilia are commonly seen on multi-ciliated cells and are known to generate fluid flow outside of the cell, such as oviduct epithelia and cell in the airways of the lung that move mucus [21].

Primary cilia, on the other hand, are non-motile cilia that act much like antennas for the cell. They receive signals from extracellular cues, both chemical and mechanical (such as extracellular flow) and stimulate many cellular responses [67]. The most well-known signaling axis coordinated by primary cilia is Hedgehog signaling, which interprets developmental cues to trigger differentiation or entry into the cell cycle [67–69]. In the absence of a Hedgehog signaling molecule, signaling receptors are excluded from the cilium. However, Hedgehog stimulation triggers translocation of receptors and downstream signaling molecules to promote Gli transcription factor activation [70–74]. Depending on the context, Hedgehog signaling can promote cell survival, differentiation, or proliferation. Proper regulation of this signaling axis is essential for organogenesis and neural patterning [47]. Mutations in cilia proteins, both those that localize to the basal body and along the axoneme, cause a variety of diseases known collectively as ciliopathies and include polycystic kidney disease, polydactyly, and Left-Right patterning defects [75].

Generally, primary cilia are features of G0/G1-phase cells in mammals and are assembled after cells exit mitosis [67]. In cells with a single cilium, the older centriole of the pair (the ‘mother’) docks at the plasma membrane to initiate ciliogenesis [20]. From there, specialized vesicles are recruited to a structure on the centriole, called the distal appendages (Fig. 1), which promote remodeling of the plasma membrane to accommodate the morphological changes necessary for cilium extension [76, 77]. Microtubules then begin to grow off of the distal end of the centriole, now referred to as the basal body, pushing the membrane outwards and filling the center of the cilium. Growth of the microtubule extension, called the axoneme, is facilitated by cilia-specific proteins allowing the centriole to preserve its identity and, when the cilium disassembles in S/G2-phase, the centriole is retained [78]. Since ciliary disassembly seems to be a prerequisite prior to mitosis, the centriole that was formerly a basal body is available to organize the centrosome during the next mitosis [67]. The centriole plays a similar role in terminally-differentiated multiciliated cells as well, although these cells have specialized centriole assembly pathways that allow for the rapid generation of hundreds of new centrioles that are competent to form basal bodies [79]. Since centrioles spawn cilia, not surprisingly, the loss of centrioles can cause ciliopathies and associated phenotypes [75]. Since the focus of this review is on centrosome-specific pathologies, ciliopathies are not discussed in depth here [reviewed in 67, 80].

While the centrosome is best known for its mitotic role in spindle assembly, the interphase centrosome plays diverse roles, particularly in specialized cell-types [18]. The multitude of functions for centrosomes seem to rely on strict adherence to the proper structure of the centriole and centrosome. As such, centrosome-associated mutations commonly alter interphase-specific centrosome functions and many of the cell-types affected by these mutations have adapted the centriole to perform specialized tasks. Therefore, identifying the etiology and pathogenesis of these diseases may reveal cell-type specific requirements for centriole structures [81].

## Functions of the mitotic centrosome

During mitosis, centrosomes assist in the construction of the mitotic spindle, a microtubule-based protein machine designed to segregate chromosomes. The spindle is a bipolar array of microtubules that interact with a plethora of motor proteins, kinesins and cytoplasmic dynein, which help power the apparatus [82–84]. Coordination of mitotic spindle function is conducted by the centrosome, without which, shape, size, timing, and the fidelity of chromosome segregation all suffer [18].

**Fig. 3:**
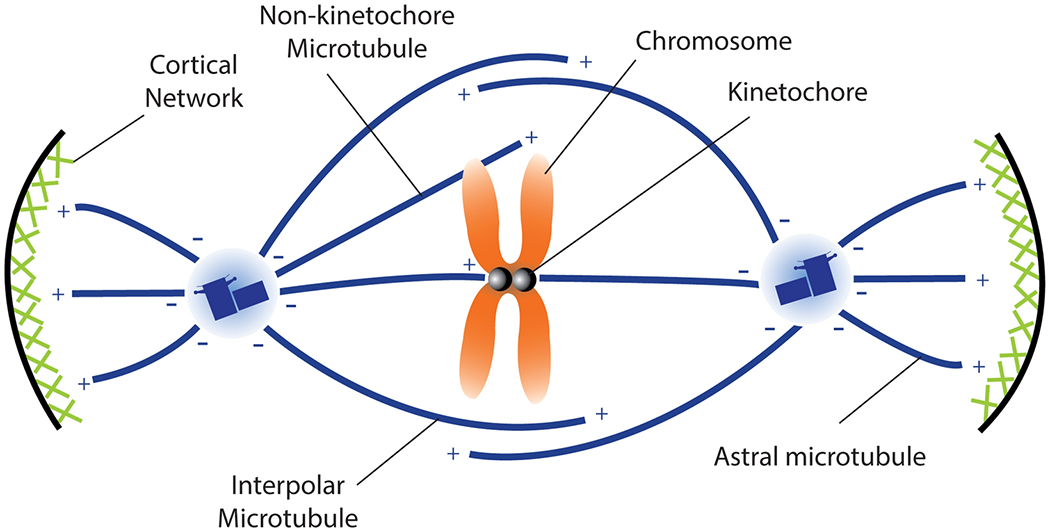
The mitotic spindle is composed of different populations of microtubules. Many, but not all, of these microtubules are generated by centrosomes which are attached to the spindle poles. Spindle microtubule minus-ends are either embedded in the pericentriolar material (PCM) or bundled together and form tapered spindle poles. Spindle microtubules are also randomly nucleated in the vicinity of chromosomes as well as produced laterally along pre-existing microtubules by the augmin complex. As their name implies, kinetochore microtubule plus-ends bind kinetochores in an ‘end-on’ attachment, which promotes their stability by suppressing plus-end disassembly. Less stable microtubule populations include interpolar microtubules with partially overlapping plus-ends that residue in the spindle midzone. These anti-parallel regions are crosslinked by plus-end directed kinesin-5 motors that effectively push spindle microtubules apart and contribute to spindle shape. Non-kinetochore microtubules also interact with chromosome arms. Astral microtubules grow away from chromosomes and towards the cell cortex where they are tethered by cytoplasmic dynein and other microtubule-binding proteins. Astral microtubules allow motors at the cell cortex to produce force and orient or rotate the spindle within the cell.

Cells normally enter mitosis with only two centrosomes and each centrosome has the capacity to organize a distinct pole of the spindle. During the beginning of mitosis, the two centrosomes separate towards opposite sides of the cell and guide assembly of a bipolar, fusiform-shaped spindle with microtubule plus-ends that partially overlap at the spindle equator and astral microtubules that interact with the cell cortex (Fig. 3). Due to the back-to-back geometry of the joined sister chromatid pair, kinetochores face opposite centrosomes and, consequently, this orientation facilitates proper amphitelic kinetochore-microtubule attachment [85]. Bioriented sister chromatids that experience an appropriate amount tension across their centromeres eventually line-up on the spindle equator and form the metaphase plate in an elaborate tug-of-war (an event known as ‘congression’). Ultimately, sister chromatids disjoin and segregate to opposite spindle poles and into newly formed daughter cells which also inherent a single centrosome. Given the ability to organize spindle poles, it is critical that cells contain precisely two centrosomes during mitosis [86]. If cells contain too many or too few centrosomes, the number of spindle poles can change, compromising the fidelity of cell division [87].

Prior to entering mitosis, the two recently duplicated centrosomes reside near one another, creating a single microtubule organizing center during interphase (Fig. 2). However, centrosomes must move away from each other and towards opposite ends of the cell to generate a bipolar spindle during mitosis [88]. This feat is primarily accomplished by the kinesin-5 motor Eg5, a bipolar, plus-end directed homotetramer with two motor domains at either end of a central rod [89, 90]. Since each centrosome nucleates microtubules with their plus-ends pointing away, kinesin-5 tetramers are optimally designed to crosslink the overlapping microtubules between them. Eg5 motor activity is slow and non-processive but, working collectively, a population of these motors can slide antiparallel microtubules apart and push their attached centrosomes away from one another [91, 92]. The importance of Eg5 can be seen when cells are treated with the kinesin-5-specific chemical-inhibitor, monastrol, which effectively blocks centrosome separation causing formation of a monopolar spindle and mitotic arrest [93].

In preparation for mitosis, centrosomes also increase their microtubule nucleation capacity by expanding the size of their pericentriolar material (PCM), a process known as centrosome maturation [18]. The expanding PCM is built around an existing scaffold that coats the outside of the centriole barrel [19]. Activation of the mitotic kinase Polo (Polo-like kinase/Plk1 in humans) at the centrosome drives maturation [94, 95]. Polo targets the centrosome scaffold and its kinase activity drives a positive feedback loop that results in rapid PCM expansion [96–101]. Increased PCM generates more binding sites for the microtubule nucleation machinery, including XMAP215, TPX-2, and NEDD1, which directly stabilize microtubules and recruit γ-tubulin ring complexes [102–106]. Together, this dramatically increases the microtubule-nucleating capacity of the centrosome during mitosis.

The high microtubule density in the mitotic spindle, partially attributed to centrosome maturation, is used to generate the forces that power mitosis. Microtubules attach to chromosomal kinetochores to align chromosomes between the two centrosomes during metaphase. Cells rely on the inherent dynamic instability of microtubules, along with assistance from molecular motors, to push and pull the chromosomes into alignment [107–109]. Kinetochore microtubules also contribute to the force production necessary to generate isometric tension across centromeric DNA. Sufficient tension on sister kinetochores is necessary to satisfy the spindle assembly checkpoint and transit into anaphase [110]. Thus, both the increased microtubule nucleation capacity and the bipolarity provided by the centrosomes facilitate efficient chromosome segregation.

Additionally, the centrosome organizes the minus-ends of spindle microtubules, helping to focus the spindle poles [111]. Most microtubules in the mitotic spindle are not directly anchored to the centrosome but rather released from the centrosome or nucleated through different mechanisms (for example, the Augmin/HAUS branching microtubule nucleation pathway) [112–116]. Minus-end directed kinesin-14 (Ned in *Drosophila*; HSET in humans) and cytoplasmic dynein motor complexes then crossbridge and move along parallel microtubules, thereby focusing spindle microtubules into a tapered pole near the centrosome [86, 112, 117–119]. Microtubule minus-end-associated proteins such as patronin (CAMSAP in humans), Mud (NuMA in humans), and Asp (ASPM in humans) concentrate at the poles and link them to centrosomes [120–122].

Although the centrosome is required for efficient mitotic progression, the fact that most kinetochore microtubules are not directly anchored at the mature centrosome seems contradictory to its necessity [114, 123]. However, electron micrographs of kinetochore microtubules suggest they are dynamically unstable at their minus ends, indicative of microtubules released from the centrosome [114, 116, 124–126]. Therefore, the increased microtubule nucleation capacity at the mature centrosome may contribute more microtubules to the mitotic spindle than is immediately apparent. Additionally, centrosome-derived microtubules act as anchor points for microtubule cross-linking, resulting in the vast majority of microtubules being physically connected to the spindle pole [126]. These interactions are suggested to provide physical stability that buffers the forces that microtubules experience [126]. Together, the diverse populations of microtubules within the spindle are used to generate pushing forces (primarily exerted by interpolar microtubules) and pulling forces (primarily provided by kinetochore microtubules) that facilitate spindle elongation and chromosome segregation during anaphase [119].

Finally, centrosomes disassemble at the end of mitosis to reestablish proper microtubule nucleation capacity and cellular organization [18]. As cells enter mitosis, kinases become activated to facilitate centrosome maturation (such as Plk1 and Aurora A) but little is known about the reverse process [100]. During mitotic exit, protein phosphatases are thought to dominate the cellular landscape and reestablish the interphase centrosome. In part, Protein Phosphatase 2A (PP2A) dephosphorylates an unknown target at the centrosome in telophase, likely PCM components Spd-2/Cep192 and/or Cnn/CDK5RAP2/Spd-5, to weaken the structural integrity of the PCM [127]. A combination of pulling forces on centrosome microtubules and endocytic trafficking machinery then remove excess PCM proteins and transport them away from the centrosome [128–131]. This leaves the centrosome with the smaller core scaffold of PCM that is maintained through interphase but act as the foundation for the next round of centrosome maturation prior to the next mitosis [18].

Just as centrosome maturation nucleates and captures microtubules at the beginning of mitosis to promote spindle assembly, centrosome disassembly promotes the reorganization of the interphase microtubule cytoskeleton [132,133]. At the end of mitosis, microtubules are released from centrosome at a rate that is sevenfold higher than metaphase release rates [133]. This release, along with centrosome fragmentation, contributes to both reduced microtubule density at and microtubule nucleation capacity of the interphase centrosome [116, 133, 134]. Although increased microtubule-nucleating activity is observed in cancer cells with an excess of centrosomes (known as ‘centrosome amplification’), it is yet unclear as to the effect of maintaining active, mature centrosomes during interphase [81].

Along with coordinating spindle assembly, centrosomes also orient the mitotic spindle within the cell, defining the cell division axis. To generate and maintain tissues, cells take cues from their microenvironment to inform when and where they must divide. As such, most cells do not divide in random directions. Instead, the mitotic spindle is oriented along a specific axis to facilitate tissue growth and decide cell fate [130, 135]. For example, cells within a planar epithelium must divide symmetrically along that plane or they will be expelled from the tissue, resulting in smaller tissues and organs [135]. Centrosomes are required to reliably orient the mitotic spindle along the designated axis.

Centrosomes not only nucleate microtubules that comprise the spindle, but they also produce microtubules that grow towards the cell periphery called astral microtubules (Fig. 3). The minus-ends of astral microtubules are anchored at the centrosome while their plus-ends interact with the cell cortex [136]. Because of this, astral microtubules can be captured by microtubule-binding proteins and motors that concentrate at the cortex, such as cytoplasmic dynein and Mud [137]. Through these interactions, spindles can rotate and reorient along the division axis. For example, in symmetrically dividing planar epithelium, cytoplasmic dynein localizes to cell-cell junctions to accomplish this task [138].

Spindle orientation can also influence cell fate decisions by promoting the asymmetric distribution of cell fate determinants. Like symmetric divisions along epithelial planes, capture and/or pulling of astral microtubules by proteins embedded in the cell cortex reposition the mitotic spindle (and thus, the cleavage plane) to produce daughter cells of different sizes and/or containing different polarity proteins and cell fate determinants [139]. Asymmetric cell divisions are a common mechanism to generate stratified epithelia and specialized cell-types, including neurons. However, the signals used to concentrate dynein generally rely on polarization cues, such as apical-basal or anterior-posterior signals to divide along a polarized axis [135].

**Fig. 4:**
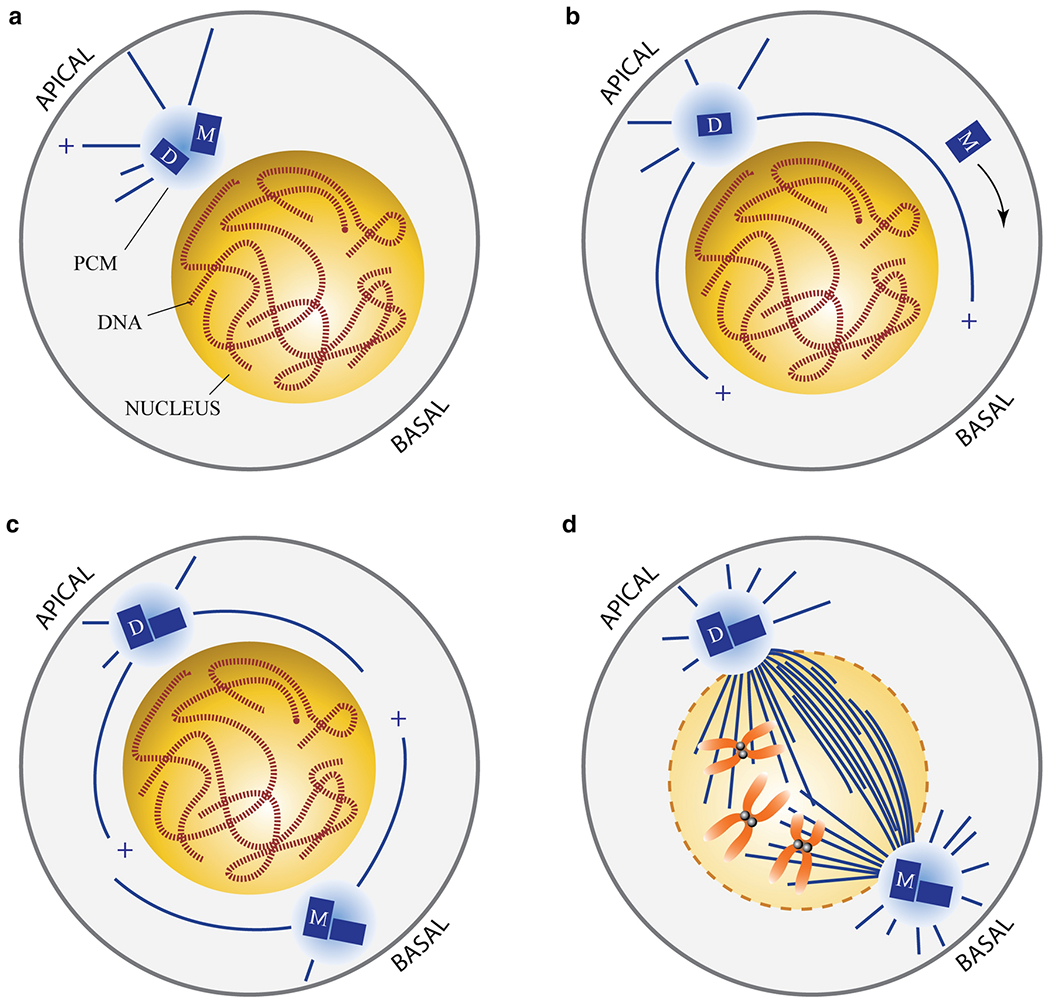
Illustration of dividing *Drosophila* neuroblast in third larval instar stage. Neuroblasts are asymmetrically dividing neural stem cells that display a non-canonical centrosome maturation cycle. **a** After dividing, the neuroblast contains a single centrosome with a mother (M)-daughter (D) centriole pair. The centrosome is tethered to the apical region of the cell through a microtubule-dependent interaction with a protein complex consisting of dynein-dynein light chain 2 (LC2)-Mud-Ana2 [145]. At this point, the mother-daughter centriole pair have physically separated (an event known as ‘disengagement’ [146]). **b** Unlike the mother centriole, the daughter contains the centriole protein Centrobin/Cbn and, along with Polo kinase, functions to retain its PCM and ability to nucleate a microtubule aster [147–150]. While the daughter centriole remains fastened to the apical cortex, the mother centriole sheds its PCM and is unable to function as a centrosome. Instead, it migrates to the basal side of the side where the mother ganglion cell cluster reside. It is unknow how the mother centriole moves to this side of the cell. Possibly is it delivered along the cytoskeletal filaments or is simply diffusion-based. **c** Both the older mother centriole and younger daughter undergo duplication in S-phase. Just prior to the next mitosis, the older mother centriole acquires the ability to recruit PCM and function as a centrosome, producing its own microtubule aster. **d** The mitotic spindle rotates and is oriented along the apical-basal axis. During anaphase, the spindle moves slightly towards the basal side of the cell, producing a small ganglion mother cell and a larger self-renewing neuroblast that retains the younger daughter centriole [149].

A classic example of asymmetric division occurs in the *Drosophila* neuroblast, a neural stem cell that delaminates from the neuroectoderm during larval brain development (Fig. 4). The neuroblast is a polarized cell-type whose apical surface sits adjacent to the neuroectoderm layer [140]. The apical surface of the cell is maintained by asymmetric localization of the Par complex: Bazooka (Baz; Par3 in humans), Par6, and atypical Protein Kinase C (aPKC) [141, 142]. Opposite that, the cell fate determinant factors Numb, Prospero, and Brat, localize to the basal side of the cell [143]. If the neuroblast divides parallel to the neuroectoderm (symmetrically), these fate determinants will be equally segregated between them, giving rise to two new neuroblasts. However, if the neuroblast divides perpendicular to the neuroectoderm, it produces one neuroblast and one ganglion mother cell (GMC); the GMC eventually becomes a neuron because inherits cell fate determinants, resulting in its cellular differentiation while the apical surface of the neuroblast is maintained adjacent to the neuroectoderm [144]. Self-renewing neuroblasts divide asymmetrically multiple times, producing clusters of GMCs at the basal domain [140].

Centrosomes are essential for efficient neuroblast differentiation. Apically localized microtubule-binding proteins capture astral microtubules from one centrosome to orient the mitotic spindle [147, 148]. Baz, a member of the apical Par complex, binds the adaptor protein Inscuteable which recruits the microtubule-binding Gαi/Pins/Mud complex [151, 152]. Mud then captures astral microtubules and anchors one centrosome on the apical side of the neuroblast [153, 154].

Interestingly, in most interphase *Drosophila* cells, centrioles recruit low amounts of PCM and, thus, do not nucleate microtubules [155, 156]. The neuroblast, however, is a unique cell-type in the fruit fly because it retains a centrosome with microtubule-organizing activity during interphase [156]. As a neuroblast exits mitosis, the centriole pair disengages and separates. Remarkably, while the older ‘mother’ centriole sheds its PCM, the younger ‘daughter’ centriole retains its PCM and associated microtubules [147, 148]. Consequently, the younger centriole (which continues to function as a centrosome) is fastened to apical side of the neuroblast through microtubule interactions with the Gαi/Pins/Mud complex, while the older centriole freely migrates to the basal cell side [109]. As the neuroblast enters the next mitosis, the basal-positioned mother centriole recruits PCM and begins to function as a centrosome again by producing microtubules and assisting in spindle formation. At the end of mitosis, the older mother centriole is deposited into the new GMC [149].

Unique mechanism in the neuroblast also contribute to inactivation of the older, mother centrosome. Instead of phosphatases, mother centrosome inactivation involves another kinase. Specifically, Polo-like Kinase 4 (Plk4) extensively phosphorylates the PCM protein Spd2, which releases Spd2 from centrioles and inactivates the older centrosome [157]. In neuroblasts expressing kinase-dead Plk4 or a phospho-null Spd-2 mutant, both centrioles retain PCM and remain active MTOCs during interphase. Consequently, neuroblasts cannot properly orient their spindle and fly brains do not develop properly [157]. Although we often simplify the centrosome’s mitotic role to spindle assembly, the centrosome orchestrates most aspects of mitosis. Because of this breadth of function, it is no surprise that the centrosome is essential for mitosis in most somatic cells. However, a major gap in our knowledge is understanding how centriole and centrosome structure contributes to each of these roles, as well.

## Centrosome instability causes chromosomal instability

Most genetic diseases caused by mutations in centrosome genes affect specialized functions in unique cell-types (e.g., neural stem cells). The centrosome is essential for efficient mitosis and development and, not surprisingly, mutations that interfere with general centrosome functions are lethal [81]. This is because genomic integrity is especially vulnerable during mitosis. Chromosomes must be equally divided into two daughter cells to avoid potentially lethal or, conversely, oncogenic errors. Dramatic centrosome dysfunction, particularly changes to centrosome number, place cells at risk of chromosome missegregation [86, 87]. As such, cells have developed extensive mechanisms to detect and respond to the mitotic errors caused by centrosome dysfunction [158]. These mechanisms police the fidelity of mitosis to eliminate cells that have experienced, or may have experienced, genomic instability.

The Spindle Assembly Checkpoint (SAC) is the best-known mitotic surveillance mechanism. The SAC prevents cells from progressing to the metaphase-anaphase transition until all chromosomes are aligned at the metaphase plate and does so by inhibiting the anaphase-promoting complex/cyclosome (APC/C) [110, 159]. The APC/C is a large E3 ubiquitin-ligase that triggers mitotic progression by targeting cyclin B1 for proteolysis and, subsequently, enables sister chromatid separation by promoting the degradation securin to liberate the key cysteine protease, separase [160]. Active separase then cleaves Scc1, a subunit of the sister chromatid-tethering cohesion complex, resulting in chromosome disjunction and separation [161]. Unattached kinetochores trigger the SAC by activating Mad2 which binds and sequesters the APC/C activator, Cdc20 [162]. Once all chromosome pairs are attached to spindle microtubules and their kinetochores are under proper tension, Cdc20 is freed to activate the APC/C [163, 164]. Normally, the SAC protects genomic integrity by providing cells with sufficient time to correct improperly attached kinetochores before completing mitosis [110]. In the context of centrosome dysfunction however, the SAC can prevent mitotic progression when chromosomes are improperly attached to an abnormal spindle.

Centrosomes are paramount to efficiently generating a bipolar mitotic spindle but are not absolutely required [164]. Alternative microtubule nucleation mechanisms exist, both at chromosomes and through microtubule branching, which can generate a microtubule network capable of self-assembling into a mitotic spindle [112, 165, 166]. However, bipolar spindle formation is dramatically stalled in somatic vertebrate cells that lack centrosomes [164, 165]. Therefore, the SAC protects cells without centrosomes from inefficient spindle formation by slowing mitotic progression until all chromosomes experience sufficient tension. Without this vital sensor, chromosome missegregation would ensue, producing chromosomal instability (i.e., changes in chromosome number and/or structure) [166].

The SAC also protects cells from mitotic defects caused by having too many centrosomes, a phenomenon called ‘centrosome amplification’ and an emerging hallmark of cancer cells [168]. Each of these supernumerary centrosomes can nucleate microtubules and organize a spindle pole during mitosis. As cells with centrosome amplification enter mitosis, kinesin-5 motors push centrosomes apart, producing spindles with too many poles (called a multipolar spindle) [169]. Chromosomes can then be captured by any of these spindle poles, sometimes even more than two; such abnormally attached chromosomes are subject to improper forces and, thus, struggle to satisfy the SAC. In some cases, cells undergo a multipolar cell division resulting in three or more daughter cells, which are typically inviable [169]. In this way, the SAC is the first line of defense against defects in spindle shape caused by centrosome amplification (or loss) and the consequent chromosomal instability that is produced.

Even though the SAC is intended to hold cells in mitosis until problems in kinetochore attachment and centromeric tension are resolved, cells do not remain in mitosis indefinitely. Instead, cells that spend too long a time in mitosis experience different fates, including delayed mitotic-linked cell death or mitotic slippage (a scenario where cells exit mitosis without completing chromosome segregation) [170]. Mitotic slippage not only doubles cellular ploidy but the number of centrosomes as well [171]. Interestingly, cells that exit mitosis after a prolonged mitotic delay are not out of trouble, as they are susceptible to G1-phase arrest and apoptosis [170]. The mechanisms that trigger this arrest in daughter cells is poorly understood but it is linked to the amount of time spent in the previous mitosis, an effect mediated through p53 [172]. On average, cultured human cells that remain in mitosis for more than 84 minutes trigger this checkpoint, even if they successfully undergo cell division. For reference, normal cultured human cells spend closer to 30 minutes in mitosis [173].

**Fig. 5:**
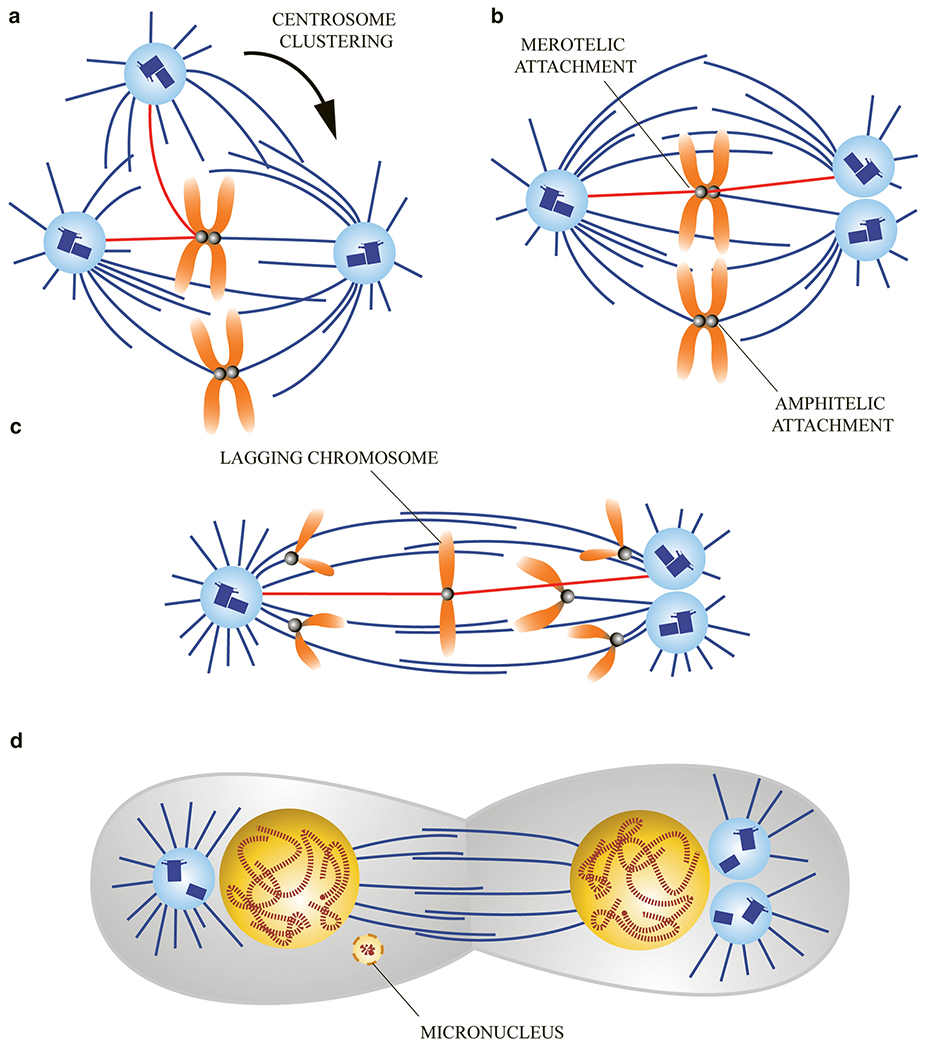
Illustration of mitotic centrosome clustering and the chromosomal instability it can cause. **a** Mitotic cells containing two or more centrosomes (amplification) form a transient intermediate structure known as a multipolar spindle. Each centrosome can form a distinct spindle pole and form end-on attachments at kinetochores (red lines). Minus-end directed microtubule-based motor proteins crosslink and slide antiparallel microtubules emanating from centrosomes and drive there clustering. In the example shown, the extra centrosome makes an attachment with a kinetochore facing the left spindle pole, but then moves to the right spindle pole. **b** Centrosome clustering allows spindles to assume a bipolar fusiform shape (known as a ‘psuedo-bipolar spindle) that is capable of segregating chromosomes. Whereas most chromosomes form proper ‘amphitelic’ kinetochore attachments (kinetochores on a sister chromatid pair are attached to opposite spindle poles), kinetochores linked to clustered centrosome sometimes form merotelic attachments (one kinetochore is attached to opposite spindle poles). Such improper attachments are dangerous as they undergo sufficient tension to satisfy the Spindle Assembly Checkpoint (SAC) and progress to anaphase. **c** Whereas most sister chromatids disjoin and segregate to opposite sides of the side, merotelic attached chromatids are trapped in the central spindle forming a ‘lagging chromosome’. **d** Lagging chromosomes are randomly segregated into a daughter cell, often producing whole chromosome aneuploidy. Even if a chromosome segregates into the proper daughter cell, there is an additional problem because, instead of decondensing and mixing with the other chromosomes in the primary nucleus, these chromosomes form micronuclei. Micronuclear envelopes frequently rupture in the subsequent S-phase causing chromosome shattering (or chromothripsis).

While centrosome amplification can result in mitotic catastrophe or arrest, cellular mechanisms exist to circumvent the problems associated with a multipolar mitosis [174]. Multipolar spindles are transient intermediate structures as cells can cluster their supernumerary centrosomes into two distinct foci, resulting in a functional ‘pseudo’-bipolar spindle (Fig. 5) [169, 175]. Centrosome clustering is mediated by the minus-end directed motors, cytoplasmic dynein and the kinesin-14, HSET [176, 177]. Similar to kinesin-5-mediated centrosome separation, minus-end directed motors crosslink and slide antiparallel microtubules emanating from two centrosomes, but slide them in the opposite direction to pull centrosomes together and drive their coalescence [176, 177]. Not surprisingly, cancer cells with supernumerary centrosomes commonly have high levels of HSET and a robust ability to cluster excess centrosomes [178]. For example, 45% of MDA-MB-231 breast cancer cells display centrosome amplification (>2 centrosomes), but only undergo a multipolar division approximately 5% of the time, due to their efficient clustering mechanism [169].

Although pseudo-bipolar spindles generate viable daughter cells, they are extraordinarily prone to chromosome segregation defects, resulting in whole chromosome aneuploidy [174]. As a multipolar spindle makes contacts with chromosomes, microtubules emanating from multiple centrosomes can connect with the same sister kinetochore. When centrosomes cluster, they can move to either side of the spindle, resulting in a merotelic kinetochore attachment (one kinetochore is attached to opposite spindle poles) (Fig. 5) [169, 175]. During anaphase when most sister chromatids disjoin and move towards opposite side poles, merotelicly-attached chromatids become trapped in the central spindle in a ‘tug-of-war’ and these are referred to as lagging chromosomes [179]. During cytokinesis, lagging chromosomes are randomly segregated into a daughter cell, potentially resulting in the production of two aneuploid daughter cells [180, 181].

Even if a lagging chromosome is segregated into the proper daughter cell, cells face new dangers. Due to its late arrival, lagging chromosomes may end up decondensing at regions too distant to allow incorporation into the primary nucleus of the cell. Instead, the single chromosome will form its own nuclear envelope, known as a micronucleus, which are common in cancer cells [182]. Micronuclei contain some of the same protein components as the nucleus but are less likely to have the correct ratio of these components [183]. Consequently, micronuclear import/export, transcription, and DNA replication are often defective and, as a result, the chromosome can experience genotoxic stress [182–184].

This is further compounded by the fact that the micronuclear membrane is more likely to rupture due to a reduced integrity of the nuclear lamina [183]. As the cytoplasm rushes into the micronucleus, slowly replicating DNA can experience double-stranded breaks [182, 185, 186]. In fact, chromosome shattering and subsequent restitching, called chromothripsis, is also common in cancer [182, 187, 188]. Chromothripsis gives rise to a several different structural alterations, including random chromosomal rearrangements, duplications, and deletions [187]. These genomic rearrangements can give rise to oncogenic translocations, loss of heterozygosity at deleted loci, and breakage-fusion-bridge cycles due to improper restitching of telomeres [189].

## Mechanisms selecting against centrosome instability

There is a growing body of evidence describing the prevalence of centrosome defects and their pathological consequences. Therefore, we find it prudent to establish the term ‘centrosome instability’ which describes the pathognomonic state of centrosome dysfunction, such as a divergence of centrosome structure and copy number from normal, healthy cells. As previously described, centrosome instability gives rise to many forms of chromosomal instability. Notably, cells have evolved p53-dependent mechanisms to indirectly identify centrosome instability due to the associated genomic damage this causes [190]. In response to most forms of DNA damage, protein levels of p53 increase either by inactivation from the E3 ubiquitin-ligase, Mdm2, or through direct phosphorylation [191]. Since p53 is a transcription factor, its stabilization also results in translocation to the nucleus. Depending on the type of damage, extent of damage, and the length of time it takes to repair this damage, p53 promotes the expression of genes that can either slow the cell cycle, promote cell cycle arrest and senescence, or induce apoptosis [192]. Because cells with centrosome defects have a propensity for chromosomal instability, p53 ensures centrosome homeostasis [190].

For example, centrosomes can be experimentally eliminated by treatment with a reversible chemical-inhibitor of Plk4 called centrinone [123]. Plk4 is the master-regulator of centrosome assembly and its inhibition prevents further centrosome duplication [193]. Over time, Plk4 inhibition generates a large population of cells without centrosomes by effectively diluting centrosome-containing cells from a dividing population [123]. Whether cells continue to divide however, depends on their p53 status. Without centrosomes, cells spend a long time in mitosis [164]. Whereas cells with intact p53 frequently display G1-phase arrest after a prolonged mitosis, p53-deficient cell lines continue to progress through the cell cycle, resulting in a proliferating acentrosomal cell line. Washout of centrinone triggers massive Plk4 activation and *de novo* centrosome assembly (a non-canonical assembly pathway that does not rely on centriole formation from existing centrosomes), which can generate a subpopulation of cells with centrosome amplification [123]. This dramatic change in copy number is a prime example of the plasticity associated with centrosome instability. In cell lines that maintain a normal number of centrosomes, cells with centrosome amplification are quickly removed from the population. Fascinatingly, when certain cancer cells lines that stably maintain an abnormally high number of centrosomes are exposed to a centrinone treatment and washout regimen, the population does not correct to a normal centrosome number. Instead, the dividing population eventually returns to their abnormal basal level of amplification and appear the same as the untreated control cells [123, 194]. At present, it is a mystery how cells can establish and maintain memory of abnormal centrosome copy number [194].

Interestingly, prolonged mitosis is sufficient to induce a p53-mediated arrest, even if cells do not experience DNA damage [173]. However, other than requiring p53, the mechanisms that mediate this arrest are different depending on whether cells display centrosome loss or amplification [158]. Cells without centrosomes that undergo a prolonged mitosis may arrest in G1-phase of the next cell cycle, and the chance of arrest increases with each consecutive prolonged mitosis [173]. Genome-wide CRISPR screens revealed that cell cycle arrest requires the deubiquitinase USP28 and p53-Binding Protein 1 (53BP1) to, somehow, activate p53 and trigger p21-mediated cell cycle arrest [195–197]. This phenomenon has been named the ‘mitotic surveillance pathway’. Conversely, cells can also arrest in G1-phase after centrosome amplification, however this does not require either 53BP1 or USP28. Thus, although cells respond the same way (specifically, G1-phase arrest), the mechanisms responding to changes to centrosome number are different and not currently understood.

As previously discussed, centrosome instability promotes the formation of micronuclei that are prone to rupture and the chromosomes they contain can experience chromothripsis [182, 183, 187]. Not only does this cause genomic damage, but cytoplasmic DNA is an innate immune trigger commonly used to identify viral infection [198]. Double-stranded DNA viruses in the cytoplasm activate the cGAS-STING pathway, leading to cellular senescence and secretion of pro-inflammatory cytokines [199]. This pathway is also co-opted by the cell to sense micronuclear rupture [198]. Upon binding DNA, cGAS generates cyclic GMP-AMP (cGAMP) dinucleotides. cGAMP then binds STING, forcing it to dimerize and activate noncanonical NF-κB pathways, including expression of pl6 which leads to senescence [198, 200]. Additionally, senescence-associated secretory protein (SASP) cytokines, such as IL-6 and IL-12, are produced, priming nearby cells to undergo senescence [199]. SASP production *in vivo* has been linked to many aging phenotypes and is generally thought of as deleterious to the health of tissues [201].

Notably, centrosome amplification itself has been linked to a novel secretome. When naive cells are cultured in conditioned media from cells containing centrosome amplification, they become more invasive [202]. In the context of p53-deficient cancers, this secretome promotes an invasive behavior in cells that contain normal centrosome numbers if they are in the vicinity of a cell that has supernumerary centrosomes. Including SASP production, these studies indicate that cells with centrosome instability can use paracrine signaling to influence neighboring cells before their ultimate demise [198, 202].

When considering the importance of the centrosome, its presence is not enough. Proper regulation of centrosome number and architecture underlie all of its functions. Although we do not know the full consequences of centrosome structural aberrations, mutants that subtly alter its structure are implicated in disease and result in impaired viability [81]. Furthermore, the existence of diverse centrioles in different cell-types suggest that centriole plasticity is essential for proper development and cellular homeostasis [15]. Understanding the diversity of centriole assembly outside of commonly used model systems will be a significant challenge for the field.

## Centrosome instability in disease

Since the first connection of centrosomes and cancer by Boveri in the early 1900’s, associations between centrosome instability and cancer have abounded [8]. Although these associations are common, models of centrosome instability over the past couple decades have given us mixed results regarding their link to cancer. Before discussing, it is important to understand the impact of the centrosome during development in metazoans.

Many studies in cell lines have shown the crucial role of the centrosome in mitosis, giving rise to the notion that centrosomes are required for life. However, a landmark study in *Drosophila* testing this question yielded surprising results: flies without centrosomes are viable and even advance to adulthood, although they died soon after hatching because they lack mechanosensory cilia [203]. Moreover, within certain tissues, the absence of centrosomes caused mitotic errors, DNA damage, and apoptosis [204]. Although cell division in these flies was slow, error prone, and asymmetric cell division was unreliable, flies were viable. The story, however, is complicated by the fact that *Drosophila* embryos contain a maternal load of proteins, capable of assembling enough centrosomes to support the animal through early development. Not until the larval stages do these mutant flies actually lack centrosomes [203]. Although unexpected, these findings challenged the dogma regarding the essentiality of centrosomes.

Additional work using the fruit fly model focused on examining the impact of centrosome amplification using transgenic animals overexpressing Plk4 [205]. Remarkably, flies tolerate centrosome amplification without acquiring large-scale aneuploidy, producing generations of such mutant flies. This study revealed that fly cells possess a robust centrosome clustering mechanism, and coupled with a strong SAC, ensures mitotic fidelity and viability [205]. This model however allowed the researchers to address a question originally proposed by Boveri: is centrosome amplification sufficient to cause tumor formation?

Whereas flies without centrosomes appeared morphologically similar to wild type flies, centrosome amplification caused hyperplastic expansion of neuroblasts [205]. However, this increase was slight and did not result in the development of large brain tumors in the mutant flies. Instead, the researchers took larval brain tissue from centrosome amplified hosts and transplanted them into the abdomen of a naive fly. Normal brain tissue can survive in the abdomen of flies without over-proliferating. In contrast, larval brains with centrosome amplification continued to proliferate, forming tumors in the fly’s abdomen [205, 206]. Additionally, the researchers observed a few cases of metastasis to distant tissues such as the eye [205]. This was the first direct evidence showing centrosome instability was sufficient to initiate tumor-like growth.

Not surprisingly, mouse models of centrosome amplification were more difficult to generate; similar to fly models, mouse models use transgenic Plk4 overexpression to achieve centrosome overproduction in cells. Unlike the fly, mouse models of centrosome amplification corroborated the original notion that centrosome homeostasis is required for proper development, as most mice die during development or shortly after birth. Surprisingly though, these initial studies found that centrosome amplification was not sufficient to form tumors [207]. Instead, the first models of centrosome amplification displayed high levels of cell death due to severe aneuploidy. For instance, centrosome amplification in the developing brain caused reduced brain size (microcephaly) and neonatal death of the mice. Even when Plk4 was overexpressed in the brain of p53-deficient mice, they did not develop brain tumors, but instead showed progressive neural degradation due to aneuploidy in progenitor cell populations [208].

Other mouse models of Plk4 overexpression showed of much the same. Plk4 overexpression in the developing epidermis was well tolerated. These cells continued to divide, albeit at a lower rate, and maintain the tissue [209]. However, a follow-up study using a similar epidermis model, showed that most mice died soon after birth because skin could not develop properly to support barrier function [210]. In mice that did survive, epidermal stratification was delayed due to p53-dependent and independent apoptosis in progenitor cells. In order to improve viability and access tumorigenicity, transgenic mice were crossed to conditional p53 knockout mice that lacked p53 expression in the epidermis. Notably, the authors discovered that transient centrosome amplification in the skin during development led to tumor formation in almost 100% of mice. As a caveat, p53-deficient mice already had a high rate of tumor formation (50% of mice), however centrosome amplification dramatically accelerated tumor onset [210]. Taken together, all signs pointed towards centrosome amplification not being sufficient to initiate tumorigenesis in mice. Possibly, centrosome amplification had exacerbated chromosomal instability in already genomically-vulnerable cells.

Other investigators were undeterred however by these results and continued to pursue the question of whether centrosome amplification promotes tumor formation. The definitive answer arrived in a model where centrosome amplification was induced in cells of adult mice. Using a doxycycline-inducible promoter, long-term Plk4 overexpression was driven within already developed tissues [211]. Remarkably, centrosome amplification caused spontaneous tumors to grow in mice with intact p53. Tumors appeared in approximately 80% of mice and cells within these tumors were highly aneuploid. So, why did these mice form tumors when other models of centrosome amplification caused cell death in tissues? The authors speculate that transgenic Plk4 was expressed at lower levels within their model, causing only a modest increase in centrosome numbers. Perhaps centrosome clustering in dividing cells could tolerate having one or two extra centrosomes during mitosis, but excessive centrosome amplification in other transgenic models was insurmountable and caused a strong p53 response which comprised cell survival.

In addition to chromosomal instability, supernumerary centrosomes promote invasive behavior and metastasis in cancer cells. When cells produce too many centrosomes, this apparently increases the microtubule nucleation capacity of each centrosome, which promotes Rac1 activation an invasive cellular behavior [212]. Furthermore, chromosomal instability due to mitotic dysfunction is sufficient to promote metastasis by increasing the micronuclear burden. In turn, micronuclear envelopes rupture, exposing DNA to the cytoplasm and activating the cGAS/STING pathway to promote transcription of an inflammatory response and metastasis [213].

Centrosome amplification is not only sufficient to promote pathological phenotypes in the laboratory, examples of centrosome amplification can be seen in nearly all spontaneous solid tumors and blood cancers [167]. Additionally, many anti-mitotic treatments have the unwanted side-effect of generating polyploid cells that possess excess centrosomes [87]. Remarkably, centrosome amplification is not the only centrosome dysfunction associated with cancer. Recent work has discovered the opposite scenario of centrosome loss in localized prostate cancer [214]. In fact, centrosome loss can generate the identical forms of chromosomal instability caused by centrosome amplification, including whole chromosome aneuploidy, the formation of micronuclei, and chromosome shattering [164, 123, 214–216]. Moreover, centrosome loss can generate sufficient chromosomal instability to transform non-tumorigenic immortalized prostate epithelial cells, capable of forming xenograft tumors in mice [214]. However, models of centrosome loss in mammals have not explored tumorigenesis due to embryonic lethality and it remains to be determined whether centrosome loss is as widespread in cancers as centrosome amplification [217–219].

While changes to centrosome copy number are common in cancer, mutations that effect centrosome function have also been found in individuals with developmental disorders. Mutations in core centriole, cilia, and PCM genes are associated with microcephaly, lissencephaly, polydactyly, primordial dwarfism, and many others [67, 81]. Genetic mouse models that examined loss of patient-related centrosomal and cilia proteins have found that many of these mutations are directly causal. Most mechanisms of disease arise because these mutant mice struggle to generate specific sets of neurons from neuronal progenitor cells. For instance, Cep63-deficient mice that show a reduced ability to duplicate centrosomes experience mitotic errors in neuronal progenitors, causing p53-dependent apoptosis and microcephaly [220]. Conversely, Cep83-deficient mice cannot properly anchor centrosomes to the apical cell surface in brain cortex progenitor cells and, consequently, these cells undergo excessive symmetric cell divisions. As a result, too many progenitor cells are produced but not enough cortical neurons, which manifests in smoothening of the brain cortex (aka, lissencephaly) [221].

It remains unclear why neural development is so sensitive to centrosome dysfunction, while other tissues remain unaffected. Possibly, neuronal cells and brain tissue require specialized centrosome functions. Conversely, there may be less selective pressure against these specific mutations. It follows that mutations affecting all cells, including the germ line, would be subject to more stringent evolutionarily pressure. This may also explain why there are no specific centrosome mutations that predispose an individual to cancer. Mutations that affect mitotic functions of the centrosome would obviously be lethal and, therefore, selected against. Mammalian models of centrosome amplification point to this idea, as the offspring are non-viable [210].

## Perspective and Pressing Questions

While many seminal studies over the past 30 years have elucidated a myriad of centrosome functions in regulation of the microtubule cytoskeleton, recent discoveries highlight emerging functions of the centrosome as a hub of other biological processes. For instance, centrosome amplification can interrupt autophagosome trafficking, disrupting autophagy and preventing degradation of autophagic targets [222]. Conversely, disrupting selective autophagy of centrosome components – a process known called doryphagy – causes centrosome fragmentation and disruption [223]. Additionally, the centrosome has recently been implicated in organization of the actin cytoskeleton, including concentrating the actin regulator LIM Kinase 1 during mitosis [224, 225]. These novel functions only add to the list of pressing questions regarding centrosome instability.

Examples of centrosome instability are becoming more prevalent in a wide gamut of diseases; however, it is unclear how centrosome instability arises. While we see changes to centrosome number in nearly every major cancer type, the mechanisms that drive changes to centrosome number remain a mystery. Perhaps centrosome instability in normal tissue is more common than we appreciate, but these cells are simply removed from normal populations through the many levels of selective pressure previously discussed. Theoretically, however, centrosome instability should be detrimental to genome integrity even in cancer cells. So, are there mechanisms that exacerbate centrosome instability within tumors (i.e., microenvironmental, inflammatory, or other cell-extrinsic factors)?

Changes to centrosome copy number only effects a small subset of cells within a tumor [87]. So, how does centrosome number heterogeneity effect that tumor? While previous work identified a pro-migratory secretion phenotype associated with centrosome amplification, the consequence to patients is yet unclear [202]. However, tumors with high genomic heterogeneity are generally more aggressive [226]. Since centrosome instability promotes chromosomal instability, tumors with high centrosome copy number heterogeneity should have high genomic heterogeneity and, thus, should also be more aggressive. Therefore, can we identify those patients with high centrosome copy number heterogeneity as a proxy for genomic instability to aid prognosis? Or could centrosome instability provide a targetable vulnerability for treating genomically unstable cancers? One recent example of this has shown that centrosome amplification sensitizes cells to autophagy inhibitors [222]. Therefore, new combination drug targets may emerge as new centrosome functions or disease roles are discovered.

## Data Availability

Not applicable.
